# Intelligent 3D Perception System for Semantic Description and Dynamic Interaction

**DOI:** 10.3390/s19173764

**Published:** 2019-08-30

**Authors:** Marco Antonio Simoes Teixeira, Rafael de Castro Martins Nogueira, Nicolas Dalmedico, Higor Barbosa Santos, Lucia Valeria Ramos de Arruda, Flavio Neves-Jr, Daniel Rodrigues Pipa, Julio Endress Ramos, Andre Schneider de Oliveira

**Affiliations:** 1 Graduate School of Electrical Engineering and Computer Science (CPGEI), Federal University of Technology-Paraná (UTFPR), Avenida 7 de Setembro 3165, Curitiba 80230-901, Brazil; 2 CENPES, Rio de Janeiro 21941-915, Brazil

**Keywords:** perception, PointCloud, prediction and object recognition

## Abstract

This work proposes a novel semantic perception system based on computer vision and machine learning techniques. The main goal is to identify objects in the environment and extract their characteristics, allowing a dynamic interaction with the environment. The system is composed of a GPU processing source and a 3D vision sensor that provides RGB image and PointCloud data. The perception system is structured in three steps: Lexical Analysis, Syntax Analysis and finally an Analysis of Anticipation. The Lexical Analysis detects the actual position of the objects (or tokens) in the environment, through the combination of RGB image and PointCloud, surveying their characteristics. All information extracted from the tokens will be used to retrieve relevant features such as object velocity, acceleration and direction during the Syntax Analysis step. The anticipation step predicts future behaviors for these dynamic objects, promoting an interaction with them in terms of collisions, pull, and push actions. As a result, the proposed perception source can assign relevant information to mobile robots, not only about distances as traditional sensors, but about other environment characteristics and object behaviors. This novel perception source introduces a new class of skills to mobile robots. Experimental results obtained with a real robot are presented, showing the proposed perception source efficacy and potential.

## 1. Introduction

Nowadays, mobile robotics is one of the most prominent research areas [1,2]. Robots with the ability to freely move through an environment, knowing their positions and able to sense the world can execute intelligent tasks. Such robots may develop tasks, for example, in industries [3,4,5] and in rescue tasks [6,7,8], or replacing humans in hazardous work.

Such robots need to perceive the environment around them to identify obstacles and execute actions without collisions. That is, the robot needs to see the world before making some decision. For this, it uses sensors that allow for converting real-world data into digital information that can be used by robots and promote actions [9,10].

There are numerous types of sensors for mobile robotics. Some are popular due to their low cost and easy data manipulation, such as color image (RGB image) provided by cameras. These sensors can be used to identify Augmented Reality Tags (AR-Tags) [11,12], among other computational vision tasks [13,14,15]. Despite its worldwide use, the RGB sensor provides a simple image of the environment, without any specific characteristic that allows smart actions.

Intelligent behaviors are not achieved without a detailed understanding of the environment that allows a reliable mapping and safe obstacle avoidance. The depth sense is primordial to obtain a three-dimensional insight and execute instantaneous reactions in the presence of obstacles or planned deviations. RGB sensors provide a cloud of points (or PointClouds), which can collect spatial information from the environment. They are used in numerous works in mobile robotics [16,17,18], mainly towards obstacles avoidance and environment mapping.

However, this type of sensor provides only the notion of distance between the robot and nearby objects without identifying them. This lack of information causes the robot to consider all objects (dynamic, static, animate, or inanimate ones) in the same way. In summary, all objects are understood as a static obstacle in a scene simplification. In fact, the environment is commonly composed of a lot of moving objects, and a static sensing compromises the robot navigation since planning must consider instantaneous information that changes over time.

For example, consider a person walking inside the same room where a robot must navigate from point A to B. This robot has a depth sensor which sees all objects in the environment (including the person) and can plan the path to achieve point B avoiding all obstacles. Nevertheless, the person crosses the robot’s path, and a new path is computed to avoid collision. This process can be eternal if the person continues to move and the robot always chooses the same side to implement an avoidance maneuver. To prevent such situation, It is primordial that more detailed information about the environment (for example, person motion forecast) is available to enhance intelligent behaviors, such as [19,20,21].

In addition to RGB sensors and depth sensors, other sensors can be used to add extra information to robots, such as temperature and localization, among others. The usefulness of this additional information requires a correlation analysis with primary sensors or simply a data fusion procedure that enhances a robot’s understanding and or improves specific sensing. In fact, the use of a group of sensors does not effectively increase the robot knowledge about the environment, but only provides several independent information sources.

The transformation of raw information from the sensors into useful information for robots requires the execution of several procedures based on different techniques. Such procedures compose the so-called perception system that helps robots perform a dynamic interaction with scene elements to make inferences about features of the environment.

For instance, some papers use data from the RGB sensor to identify objects, and others combine such data with methods to segment objects in environments with 3D sensors [22,23]. These works manipulate raw sensor data to extract some useful information. The problem with this approach is that it should be done to each robot and a specific task, without any generalization.

There have been several object recognition techniques developed in recent years [24,25,26]. Deep learning techniques stand out as the best choice due to the best accuracy for object detection tasks [27,28,29,30,31,32]. Among them, one that stands out is YOLO (You Only Look Once) [33] due to its high accuracy and quickness in object recognition tasks. Such characteristics make YOLO a potential tool for being integrated into a sensor allowing a more detailed information catch.

This work proposes a novel intelligent perception source that introduces a new class of mobile robots skills through a detailed sensing of environment components. The approach rigorously decomposes RGB-D camera data through three analysis: lexical, syntax, and anticipation. Thus, decomposition is based on Semantic Description and Dynamic Interaction (SD2I). Mobile robots with SD2I perception will identify objects from the environment and understand the dynamic behavior of time-variant elements, allowing intelligent interaction.

This paper is organized as follows. A brief description of the used 3D perception sources is carried out in Section 2. The proposed perception system is developed in Section 3. Section 4 brings practical experiments and results and a discussion about the efficacy of the proposed system. Section 5 concludes the paper presenting an overview of the work.

## 2. Overview of Used 3D Perception Sources

**Figure 1:**
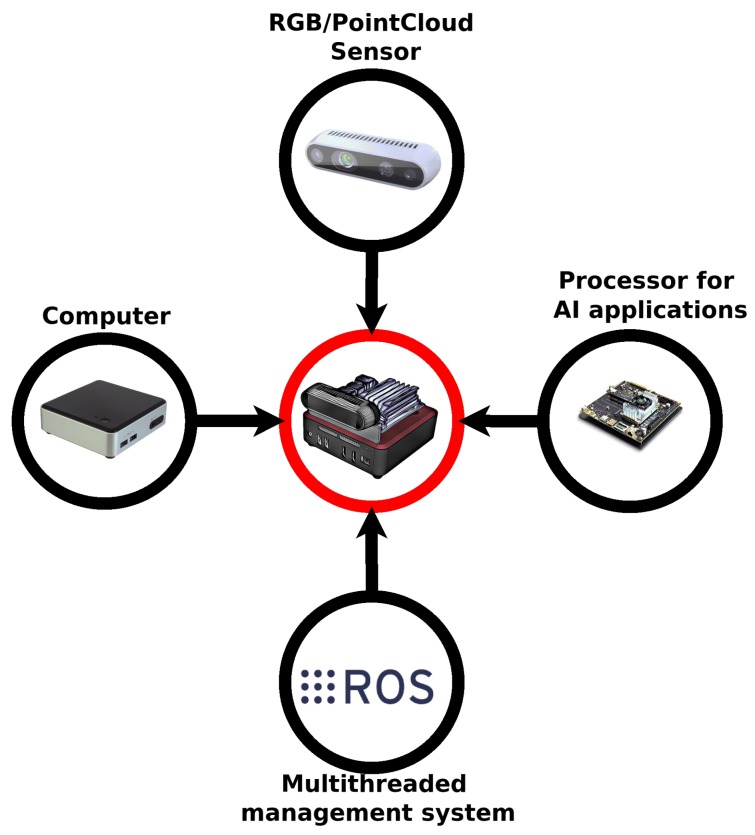
Intelligent 3D Perception (I3P) system.

The approach developed in this paper applies Semantic Description and Dynamic Interaction (SD2I) to collect information from the environment through RGB-D sensors and convert them into useful and intelligent information for a robot. In addition, we also present a compatible embedded hardware supporting such approach. An illustration of the proposed system, called the Intelligent 3D Perception (I3P) system, can be seen in Figure 1.

The SD2I methodology is based on an embedded system with a 3D sensor. The PointCloud is acquired through an RGB-D sensor, which can be Intel RealSense [34], Kinect v1 or Kinect v2 [35], among others.

I3P system uses YOLO (You Only Look Once) to identify the objects by the RGB image from the source of perception. The third version of YOLO is available since 2018 [33]. In addition to the standard version, there is the “tiny-YOLO” version, which makes it possible to identify the same objects with lower precision. The main advantage of “tiny-YOLO” is a reduced number of layers, which makes it ideal to be embedded. Moreover, YOLO has showed good results when used with several different datasets, such as COCO [36], PASCAL VOC [37], and the KITTY dataset [38]. In this way, YOLO becomes suitable for object recognition in any previously cited databases.

A robot embedded device using YOLOv3 must have processing capacity to run a Convolutional Neural Network (CNN) [29]. Such devices make use of GPUs to execute these tasks; some examples are Jetson Tx1, Jetson Tx2, Jetson Nano or Jetson Xavier [39]. For the I3P system, this device is corresponding to *Processor for Ai application* in Figure 1.

The I3P required processor must be capable of collecting information from Points Cloud and from the Processor for the AI application, processing and returning information in a time period useful to the robot. A mini computer is chosen due to its processing power since the information delay due to an RGB-D sensor affects the run time of the proposed strategy. A suggested piece of equipment is Intel Nuc I5 (Santa Clara, CA, USA) [40] or similar.

The perception sensor is a distributed system composed of two processing sources and a 3D sensor. The I3P system management is based on an *Robot Operating System* (ROS) [41] to gather all information coming from different sources. ROS is a framework with tools that allow the use of parallel programming and distributed systems. Thus, the I3P system can operate with various sources of processing, and perception, as well as providing a method for the robot to read the information from the sensor in a comfortable and agile way.

All components presented herein compose the hardware supporting the application of the approach proposed by this paper.This will be further detailed in Section 3, where the developed methodology for I3P system will be presented in detail.

## 3. Semantic Description and Dynamic Interaction (SD2I)

**Figure 2:**
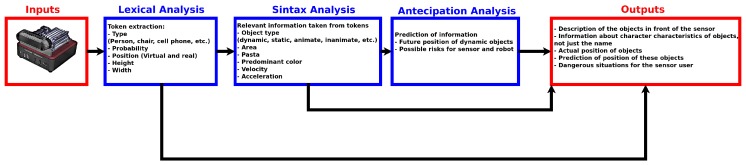
An overview of *Semantic Description and Dynamic Interaction* (SD2I) methodology.

This paper presents a new intelligent perception source with a high abstraction level to mobile robots. It is called Semantic Description and Dynamic Interaction (SD2I), and it is shown in Figure 2. The main goal is the introduction of a new class of skills to mobile robots, allowing their interaction with a large group of time-varying objects from a crowded environment. The proposed methodology is conceived to be embedded into the Intelligent 3D Perception (I3P) system described above or another similar powerfully processor.

The SD2I methodology comprises three distinct steps to process and extract the environment features. The first step is the *Lexical Analysis*. In this step, the environment objects are identified and their attributes such as kind, height, width, confidence, and position are extracted. The second part, *Syntax Analysis*, consists of an element (Token) analysis, in which all objects’ particular features like speed, acceleration, mass, and strength are estimated. Finally, the *Anticipation Analysis* uses the estimated parameters to infer future actions of dynamic objects. These analyses will be detailed in the following subsections.

### 3.1. Lexical Analysis

**Figure 3:**
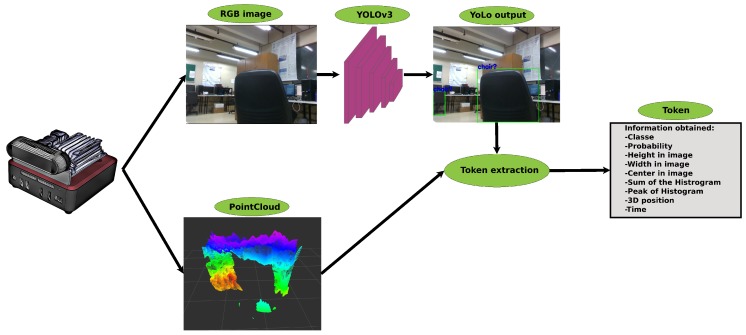
An overview of Lexical Analysis.

Lexical analysis is responsible for extracting information from the environment, generating objects (called *Tokens*) with specific characteristics. Therefore, each Token will contain various pieces of information about a single object. Figure 3 presents a diagram showing the steps of the lexical analysis.

**Figure 4:**
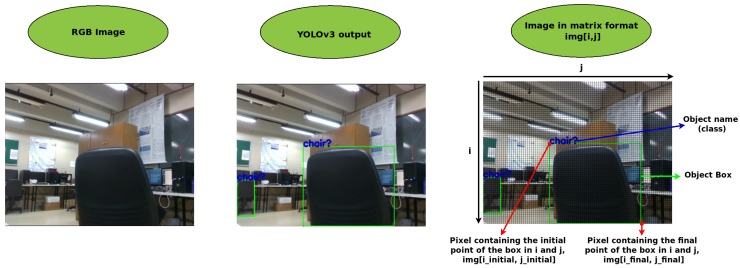
Extraction of the tokens from the image.

From Figure 3, all extracted information is associated with a Token. Such information are obtained from the RGB sensor (class, probability, center in pixel, width in pixel, height in pixel, sum of the histogram and peak of histogram) and the others come from the Depth sensor, as a spatial position of the object in the sensor frame. YOLOv3 is used for Token identification. For this, YOLOv3 has been trained with the COCO database [36] so that 80 different objects can be identified. The YOLOv3 convolutional neural network (CNN-YOLO) provides the name of the object, class, probability, and a box around the object from which the width and height are obtained. Figure 4 shows an example of data provided by the network from an image, and the strategy used to calculate height and width information.

The class of Tokens are obtained as the output of CNN-YOLO without any pre-processing step. The other data need some processing for their extraction. In the lexical analysis, the objective is only to extract the relevant data; all relevant information will be obtained from this data in the next syntax analysis step. Therefore, Token extraction must be carried out with as little computational effort as possible.

From an object image, a subtraction is made between the i_final minus the i_initial pixels, as shown in Equation (1), in order to obtain the object’s height information in pixel. The same procedure, using *j* (Equation (2)), is used to get the width. In order to obtain the central pixel of the object in the image, center_img[x,y], Equation (3) can be used. It is worth mentioning that the obtained position refers to the object in the image, not its actual location in the world, which will be obtained later. Therefore, some characteristics referring to the object and its position in the RGB image can be evaluated, for instance, height, width, and center:

(1) height_img=i_final−i_initial,

(2) width_img=j_final−j_initial,

(3) center_img=[(i_initial+(height/2),j_initial+(width/2)].

**Figure 5:**
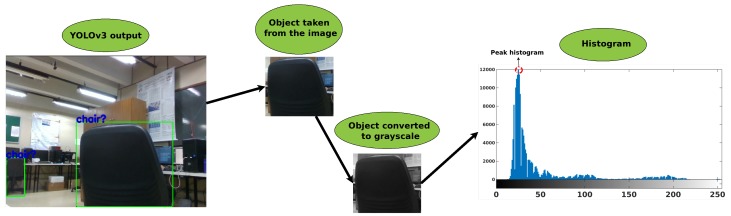
Analysis of histogram.

The predominant gray tone from an image can be captured by a histogram [42,43]. Thus, such histogram is added as an object information and its peak is saved into the Token. This information may be interesting to identify if the object is light or dark, for example. Other information taken from the histogram is the sum of all values. This information can be used to identify the same object at different time states. Only the object being observed is cut out of the image to obtain the histogram for each element. After cutting the object, the image is converted to grayscale, and then its histogram is generated. Figure 5 gives an example, where the complete image is presented, the cut object is highlighted, and its histogram identified peak is computed.

The 3D depth sensor associates each pixel of the image (img[i,j]) with a 3D point (pc[x,y,z]) of its point cloud. It is thus possible to identify the actual position of the object in the world, from the position of the object in the image. The proposed strategy consists of averaging the points relative to the object, resulting in an estimate of its position. However, the 3D sensor does not always provide the distance of all pixels; when, for different reasons, such information can not be read, the corresponding data position img[i,j] are pc[NaN,NaN,NaN] or some other invalid value. The method to avoid obtaining erroneous values consists of averaging the pixels that are in the box of the object, ignoring the values that are not valid. A second error can also happen when the box is larger than the object, generating 3D points that do not correspond to the object in question. This error is avoided with the use of only 60% of the box for averaging, ignoring the borders. The calculation of the new box consists of adding to the center of the object (center_img) 30% for more and for less of the height (height_img) for the values of *i*, and 30% for more and for less of the width (width_img) for the values of *j*.

Algorithm 1 presents the proposed strategy for identifying the object’s 3D position, where the inputs are the point cloud provided by the depth sensor, with a resolution of 640 × 640, and each position contains a 3D point [X, Y, Z], named pc[640,480,3]. The second entry consists of the center of the object in the image (center_img[i,j]). The third and fourth entries, respectively, are the object’s height and width, in pixels, provided by the image, called height_img and width_img. The computation is made by averaging in X, Y, and Z of the points in the center of the object’s box, reduced 40%. The output of the algorithm, a single 3D position [X,Y,Z], is found containing the position of the object considered by the proposed strategy, named p_center[x,y,z].

This strategy is applied to all objects identified in the image, extracting the Tokens for all objects identified by YOLOv3. To review, Tokens are object name (class), obtained by YOLOv3; height of the object in the image (height_img) is obtained by Equation (1), width of the object in the image (weidth_img) is obtained by Equation (2), central pixel of the object in the image (center_img) is obtained by Equation (3), sum of histogram (sum_histogram) and histogram peak (peak_histogram) are as seen in Figure 5 and real position of the 3D object (p_center) is obtained by Algorithm (3). The next steps convert the Tokens into smart information.

**Algorithm 1:** Identification of the object’s 3D center.

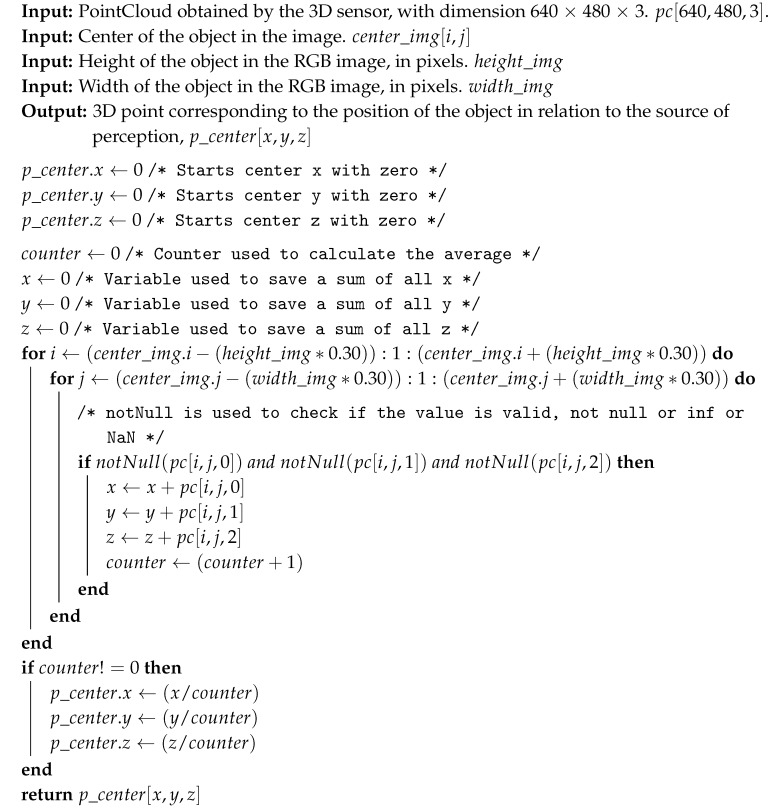



### 3.2. Syntax Analysis

**Figure 6:**
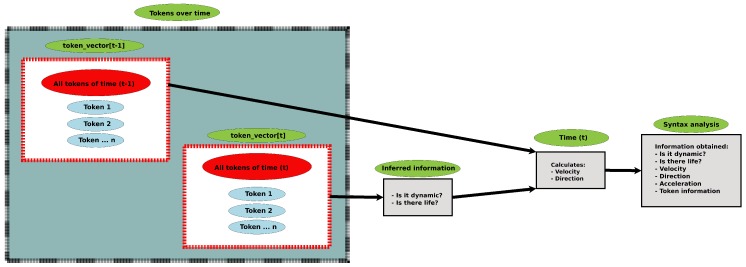
Overview of syntax analysis.

Syntax analysis aims to convert information from lexical analysis into useful information for the robot. At the end of this analysis, the object will be classified as dynamic or non-dynamic, animate or inanimate, and its speed, direction, and acceleration will be calculated if the element is time-variant. Figure 6 gives an overview of the Analysis syntax.

The objects will be divided into categories, as static or dynamic objects and animate or inanimate objects. When the objects are classified as static or dynamic objects, the robot can make use of this information to know if there is a risk of an object moving around. Knowing if the object is animate or inanimate, the robot can use this information to measure its actions, to avoid acting dangerously and risking its integrity. Table 1 presents examples of objects that belong to dynamic category and life category (animate); other objects that don’t belong to any of the two types are static or inanimate. A list of such objects can be accessed in [36].

The computation of dynamic objects’ velocity, direction, and acceleration consist of collecting the Tokens at two different time moments. The variable *t* and t−1 will be used to respectively indicate present and past into the vector of token token_vector[time][token_id].

To identify the same token at different time periods, a comparison between the data from a previous (t−1) moment and the current one (*t*) is necessary. The compared information are the token’s class (class), the peak of the histogram (peak_histogram) and Euclidean distance between the central points (p_center). First, they are compared in different moments (*t* and t−1) to verify if they belong to the same class. The second step is to use the histogram peak, where the objects are separated by gray tones, so a light grayscale dynamic object in the t−1 is compared only with the light grayscale dynamic object at the instant *t*. Finally, the Euclidean distance is used as the last criterion, considering that the tokens are generated at a small-time difference, something between 0.1 and 0.5 s, and a Euclidean distance of 0.7 m is considered as the maximum possible value for the displacement of an dynamic object of this time interval. This way, if two objects have the same class, the same shade of gray, and separated by a valid (small) value of a Euclidean distance between them, then they are the same object.

Algorithm 2 presents the procedure to identify the same object at two different instances of time. There are two vector inputs to the algorithm, where the first one contains all the present-instance tokens saved, while the other has all the past-instance tokens, the vectors are called token_vector[t].[token_id] and token_vector[t−1].[token_id], where *t* refers to the present instance time and *t* − 1 to the past instance. The time difference depends on the processing capacity of the sensor, ranging from 0.1 to 0.5 s. In the algorithm processing, a comparison between all objects of both vectors is made. Where it is verified, if both objects are dynamic, then if they belong to the same class; next, if the difference between the histogram peak is less than 30, and, finally, if the distance between their centers is less than or equal to 70 cm. If all the requirements are met, the position of the two tokens is saved in a third vector, called same_token[position].[id_(t),id_(t−1)]. In the future, these positions will be used to get the information of a Token to calculate the direction, velocity, and acceleration.

**Algorithm 2:** Identify the same object in two instants of time.

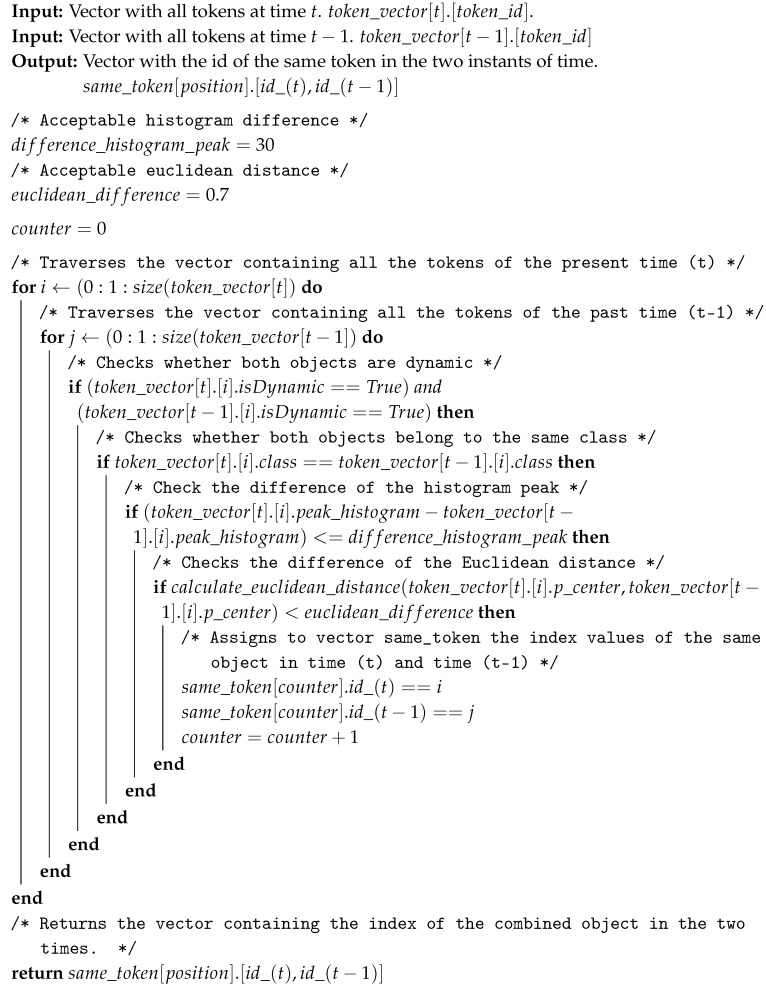



The direction of a dynamic object can be computed by the function atan2 that provides the angular difference between the two points [44]. Then, an angular deviation of 180 degrees is added to this difference, generating the course of the dynamic object. Algorithm 3 presents the complete computation of object direction. As input to the algorithm, there are the two vectors with all tokens saved, in the present instance (*t*) and in the past instance (*t* − 1), with the respective names token_vector[t].[token_id] and token_vector[t−1].[token_id]. The third input concerns the output of Algorithm 2, where the identification of the same tokens is stored, the name of this entry is same_token[position].[id_(t),id_(t−1)]. The processing consists in obtaining the angular difference between the two points representing the same token at different times so that it is possible to obtain the direction of the token. As output, a variable called as is created where the dynamic token direction, velocity, and acceleration data are stored. In this algorithm, the direction is saved in the Syntax_Analysis[id].direction field.

**Algorithm 3:** Calculate the direction of the dynamic object.

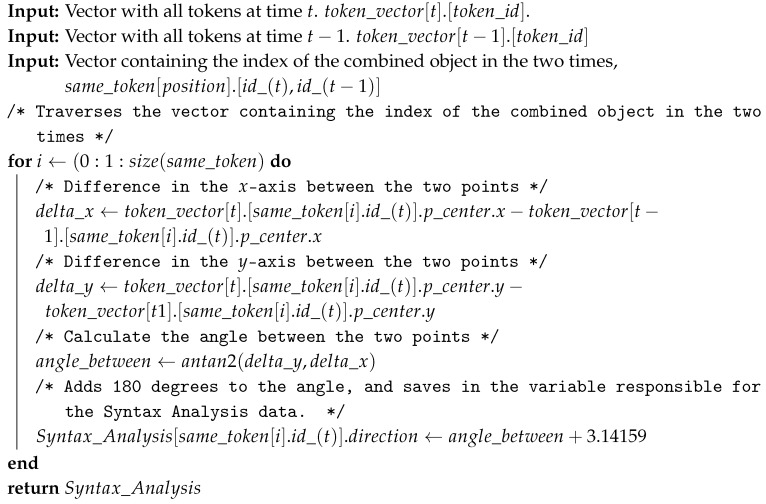



The ratio between the variation of position and the variation of time is used to calculate the velocity of the dynamic object at time t. This feature is shown in meters, and the time in seconds, so the speed obtained is in meters per second (m/s). The Algorithm 4 presents the method applied to all the dynamic points identified by the Algorithm 2. The inputs to the algorithm are the same as those in Algorithm 3, while their output is the addition of speed of the moving token, called Syntax_Analysis[id].direction. The algorithm processing consists of taking the time difference between the two tokens, the Euclidean distance traveled, and finally calculating the velocity by dividing the distance by time.

The acceleration is calculated using the speed variation divided by time. To calculate the acceleration, it is necessary that the object in question already has speed. Therefore, the velocity is first calculated, and, in the next cycle, its acceleration is calculated. Algorithm 5 brings the acceleration calculation, where the entries are the same as in Algorithms 3 and 4. The processing consists of first checking if the tokens are assigned any speed in the Syntax_Analysis[id].velocity field. Thus, the difference between the velocities of the tokens is calculated, and the time difference is obtained. Finally, acceleration is calculated by dividing the velocity variation by the time variation and saved in the Syntax_Analysis[id_(t)].acceleration variable.

**Algorithm 4:** Calculate the velocity of the dynamic object.

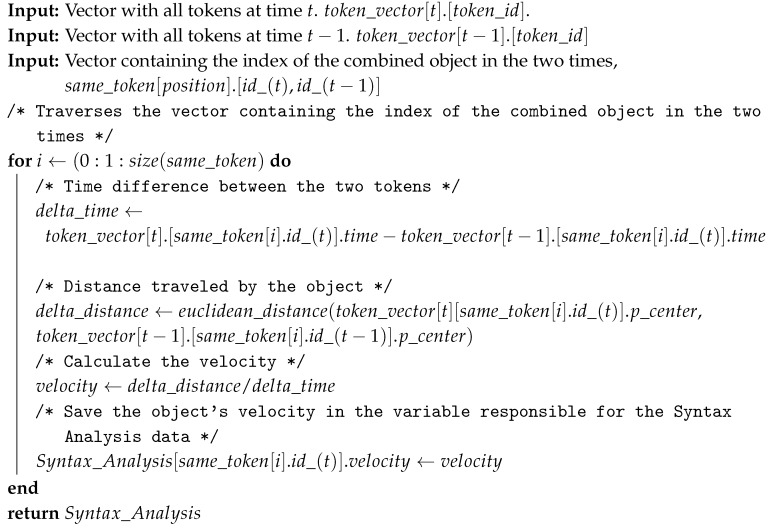



**Algorithm 5:** Calculate the acceleration of the dynamic object.

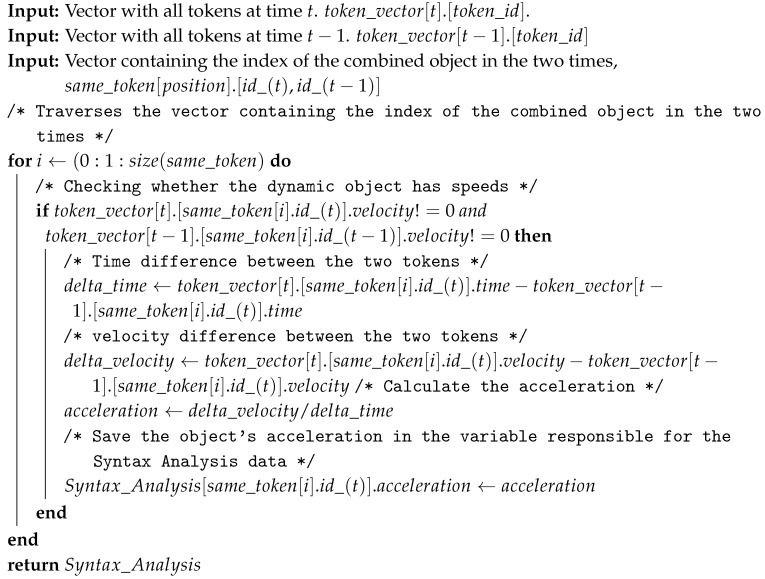



At the end of the syntax analysis, there is some new information of the token—for instance, stating if the object is dynamic or not, and whether it has a life or not (Table 1). The is also the calculation of the object’s direction (Algorithm 3), velocity (Algorithm 4) and acceleration (Algorithm 5). The next step consists of a strategy of anticipation, where the future position of the object will be calculated, based on speed and acceleration, to take preventive actions.

### 3.3. Anticipation Analysis

**Figure 7:**
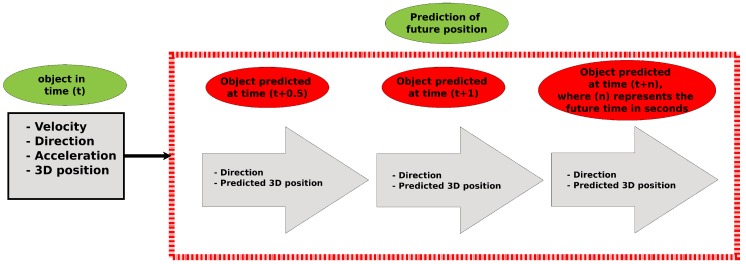
Diagram of the anticipation strategy.

The future position of dynamic objects is estimated through the data obtained in syntax analysis, in the next SD2I step, called anticipation analysis. The prediction of future behavior of time-varying elements is primordial to the robot attaining reliable path planning [45]. Moreover, for safety reasons, unnecessary calculations must be prevented during trajectory planning or maneuvers to avoid collisions with objects that are moving toward the robot. Figure 7 presents a diagram of the proposed strategy.

The object motion estimation is based on acceleration, speed, and direction of the object. It is always assumed that the element maintains its direction. The Intelligent 3D Perception is designed to predict behavior at two future times: 0.5 and 1 s ahead. Equation (4) presents the calculation used to define the object displacement in time, where time must be replaced by the time to be predicted. The variable Syntax_Analysis possesses the direction, velocity, and acceleration of the object:

(4) $$displacement=Syntax\_Analysis.velocity*time+\frac{Syntax\_Analysis.acceleration*time^{2}}{2}.$$

The predicted position is calculated by adding the displacement to the current object position, as shown in Equation (5). This process is carried out for two different time frames, being time=0.5 and time=1:

(5) $$\begin{array}{c} future\_position=current\_position+displacement\\ future\_direction=current\_direction. \end{array}$$

The next section will present the experiments and results. An explanation about the user interface detailing the displayed information is also presented.

## 4. Experiments and Results

The data provided by the proposed perception source are published in the format of ROS topics [41], where the robot can access them in an agile and easy way. This information allows the robot to make decisions and take action in navigation time. The robot accessing all the information of each Token is assured. The available information is: class, probability, center of the object in the image, center of the object in 3D, width in pixels, height in pixels, histogram peak, histogram sum, speed, direction, acceleration, whether the object is dynamic and if the object has life or not. This information is returned directly to the robot, without the need for any additional processing.

To facilitate information understanding, a graphic strategy is developed. Data can also be displayed in graphical format by using Interactive Markers [46]. This way, it is possible to view the objects in a 3D environment; additionally clicking on the object will display all collected information related to it.

**Figure 8:**
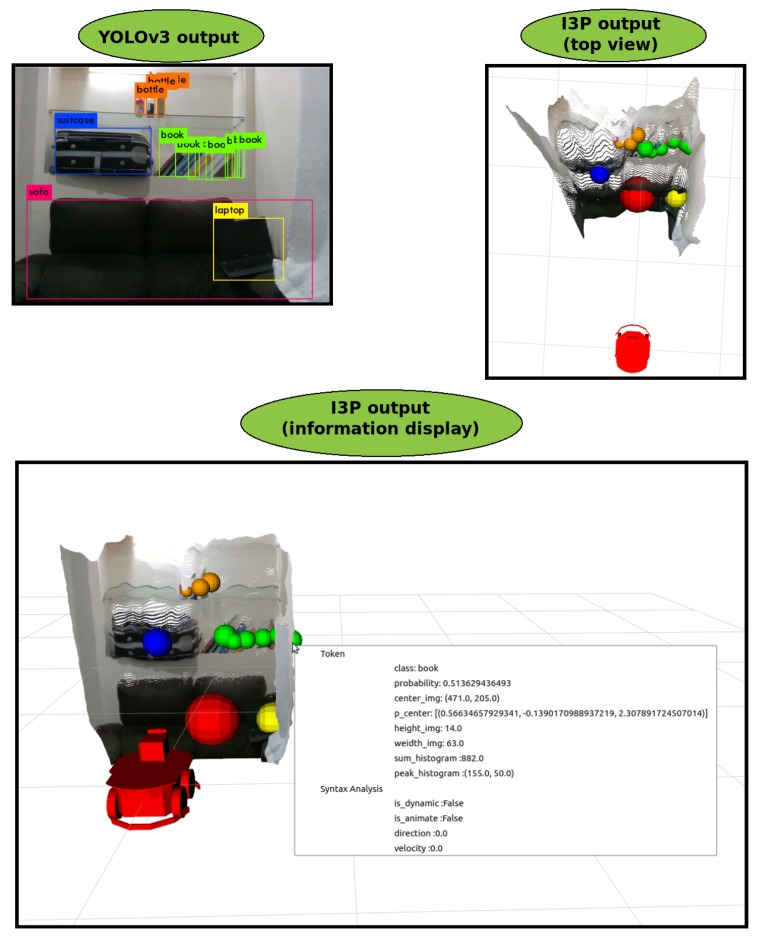
Result of the application of the Lexical Analysis.

A first experiment in which the perception source was pointed at a shelf, as in Figure 8, is carried out to validate the Lexical Analysis. Figure shows the RGB image output added to YOLOv3, (YOLOv3 output). The output of the perception source *Intelligent 3D Perception* (I3P) is presented in two ways: a top view and a frontal view. In these views, each object position is checked against the source of perception (Algorithm 1). The objects are represented by colored spheres, where yellow represents the laptop, red the sofa, green the books, orange the bottles and blue the suitcase. The source of perception I3E is represented by a red robot. In the figure, the Point Cloud with color (RGB-D data) is also displayed. All information obtained from each object can be viewed by clicking on its respective sphere. The image *I3P output, (information display)* provides an example, where all information obtained from the book object on the right is displayed. The same can be done with any identified object.

**Figure 9:**
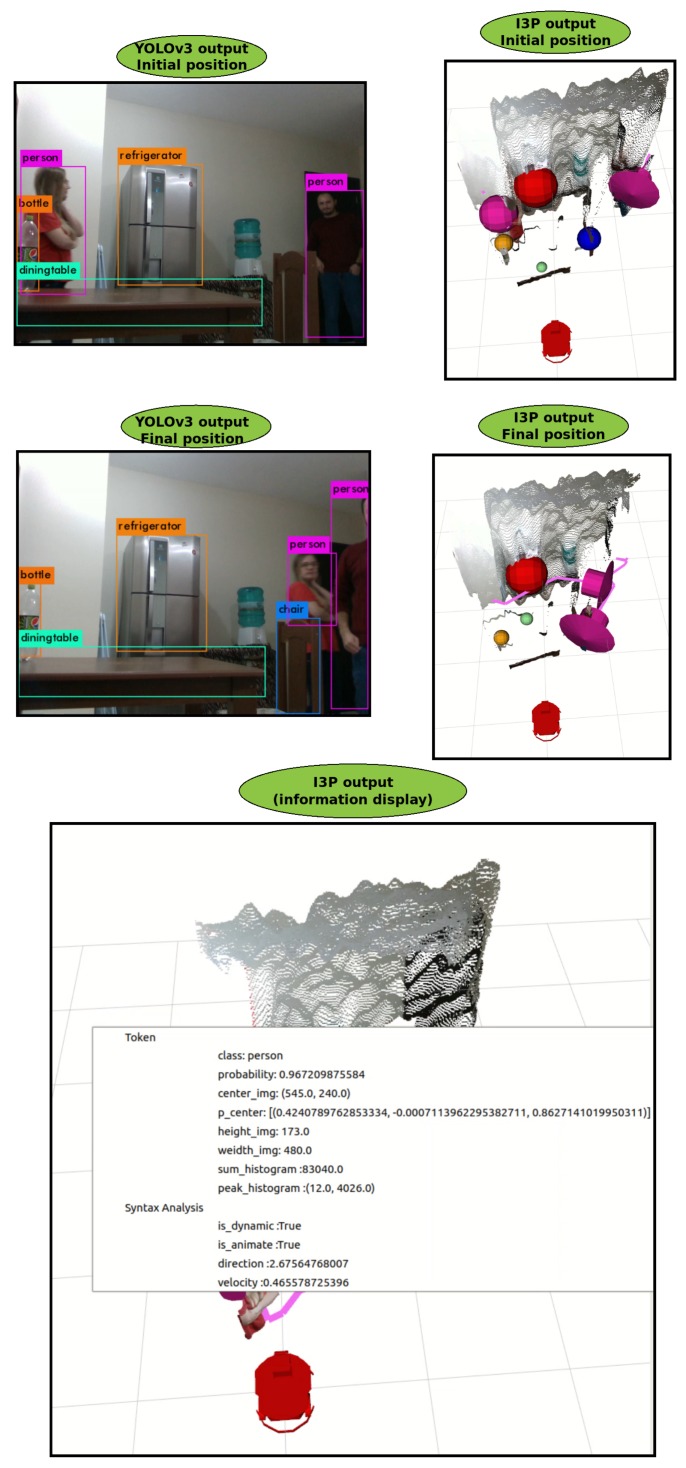
Result of the application of the Syntax Analysis.

The Syntax Analysis is concerned with obtaining information from data provided by the lexical analysis. First, the object behavior is inferred. If it is a dynamic and or animated object (Table 1), then all the Tokens are saved in two instants of different times and the procedure to identify the same object in these two instants is applied (Algorithm 2). Once this is done, it is possible to calculate its direction, velocity, and acceleration based on the displacement of the object (Algorithms 3–5). Figure 9 shows the result of the syntax analysis, when two people are moving in front of the robot. As soon as the dynamic object is identified by I3P, it is converted to a vector, which points to the direction in which the object is moving. The figure shows the path developed by the object (pink trail) so that it is possible to infer its direction, as well as validate the proposed strategy with multiple dynamic objects of the same class, one of the situations with the highest degree of complexity. In this Figure, the refrigerator is represented by a red sphere, the dining table by a light green sphere, the chair by a blue sphere, the bottle by an orange sphere, and person is represented in pink.

**Figure 10:**
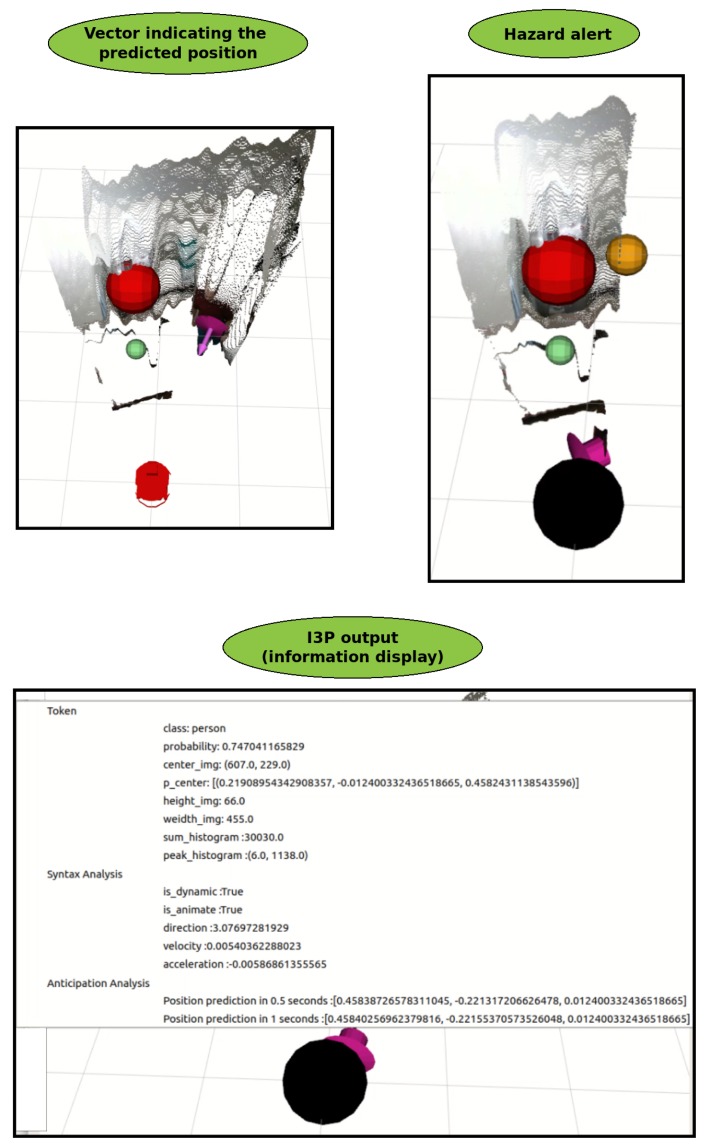
Result of the application of the Anticipation Analysis.

The Anticipation Analysis consists of predicting the future position of the moving object by its direction, speed, and acceleration. Figure 10 shows the working strategy, where the purple vector represents the future position of the object in 1 s. This time frame can be changed to identify the future position of the object; the Equations (4) and (5) are used. A second visual strategy has been developed where a black sphere is created around the perception source if the future position of a moving object may come crashing into the robot.

The proposed strategy was implemented and proved to be satisfactory for use in mobile robots. A specific sensor to provide intelligent information slows down the development of intelligent robotic systems and prevents accidents if the information provided by the sensor is used correctly, fulfilling the main objective of this paper.

### Accuracy and Precision

The error of the proposed strategy of lexical analysis corresponds to the object identification error of YOLOv3 plus the distance error provided by the depth sensor. The YOLOv3 has an accuracy of 35.4 mAP (mean Average Precision). The position error of the 3D object refers to the sensor error and may vary according to the employed perception source. The paper [47] presents the data from the RealSense D435 3D sensor, which was used by this work, which shows an error of 0.707 mm for an object at 1 m. Based on the data provided by the work, an object identified at 1 m by the strategy proposed by this paper would have an accuracy of 35.4 mAP on the object identification and an error of 0.707 mm on its position.

One of the problems that can occur when calculating velocity and acceleration is not having the same object in two moments. This issue is caused by YOLOv3 not identifying the object in a specific image frame, or the proposed strategy not recognizing the same dynamic object. Ten experiments were performed to quantify the accuracy, where a dynamic object moved around nonstop within the sensor range. During the experiment period, all Tokens data were saved. Tokens that have velocity, direction, and acceleration data are considered valid. That did not track the same objected are considered stopped were defined as errors for obtaining I3P sensor data. The results are presented in Table 2, containing the duration of each experiment, the amount of total tokens captured, the calculated tokens per second, the number of tokens with velocity, direction, and acceleration data, number of tokens with calculation errors as well as the percentage of the error. The I3P sensor error in the experiments was 4.835% on the average.

Anticipation Analysis is valid for different tasks, such as high preservation by avoiding collision with an object moving towards the robot, or for trajectory calculation. A dynamic object was taken towards the robot in a total of 10 experiments, aiming to validate the analysis in a quantitative way. Data were collected from their actual position, and position established by the Anticipation Analysis, and then the Euclidean distance from both locations to the robot was performed. An average of the variation of the two places was shown, where the obtained result was of 84 cm. On average, the robot was able to anticipate an object’s movement towards it from up to 84.79 cm. The smallest distance difference between the tests was 34.43 cm and the largest 2.77 m. This distance varies with the speed of the object.

## 5. Conclusions

The paper has presented a novel source of perception, capable of identifying objects in 3D, and extracting as much information as possible, such as name, position in the real world in relation to the source of perception, predominant gray tone, speed, direction and acceleration, in addition to predicting its future position. The proposed approach is carried out in three steps. A *Lexical Analysis* was performed, where the RGB image from the sensor was used to identify the objects through YoLoV3. From the image, data such as the class of the object, center of the object in the image, Box around the object, probability of hit and histogram peak were identified. Moreover, the Lexical Analysis also implements the computation of the object position in the real world, from its position in the image, using the cloud of points provided by the depth sensor. All these data were stored in a Token. Each Token contains information about each object identified in the scene. The error of the sensor is highly dependent on YOLOv3 accuracy, which is 35.4 mean average precision. The 3D position error from the sensor, which is specified by the manufacturer by up to 2%.

The second step consisted of a *Syntax Analysis*, where two Tokens were obtained at different time instants, called (t) for the current instant, and (t-1) for the previous instant. A procedure was then developed to identify the same Token at both instants of time. Having the same Token in both instances of time, it is possible to calculate object properties such as speed, direction, and acceleration, based on the known data of each Token. This procedure is validated through the analysis of error from obtaining the velocity, direction, and acceleration data of the dynamic objects by the strategy proposed by this paper resulting in an absolute error of 4.835%.

Finally, with velocity, direction, and acceleration, an *Anticipation Analysis* is developed, where the position of the moving object at two future instants of time was predicted. These data are important for a mobile robot in order to avoid future collisions with a moving object. In the experiments performed, it was possible to verify that an object towards the robot with an average distance of 84.79 cm, having particular cases of identifying at a distance of up to 2.77 m, makes this sensor better suited to provide information for avoidance behaviors.

At the end of these three analyses, the proposed perception source provided the robot with the ability to identify objects, know their position in the world, calculate their speed and acceleration, predict their future position and present the obtained data in an intelligent way, alerting the robot and the viewer about collision hazards.

The sensor has some particular issues to be improved, such as the instantaneous lack of calculation the speed, direction, and acceleration of dynamic objects in single frames. This problem does not pose a risk to the robot because a frame lasts, on average, 0.3367 s, and the error happens in isolated cases in very tiny time intervals. As future efforts, it is possible to refine the strategy to work with a more significant amount of time intervals, thus preventing such errors from occurring.

The use of an intelligent sensor can prevent the loss of material and processing resources. By predicting the future position of a dynamic object, it is possible to develop a high preservation strategy to avoid collisions, for example. Focusing on processing and performance of the robot, it is possible to avoid the recalculation of trajectory, considering busy positions where dynamic objects have a chance to occupy. These are just a few examples of how the proposed strategy can contribute to the advances of research and development in mobile robotics.

## Figures and Tables

**Table 1:** Inferred knowledge in objects.

	*Object Class*
***Is it dynamic?***	person, bicycle, car, motorbike, aeroplane, bus, train, truck, boat, bird, cat, dog, horse, sheep, cow, elephant, bear, zebra, giraffe, frisbee, snowboard, sports ball, skateboard, surfboard tennis racket, chair
***Is there life?***	person, bird, cat, dog, horse, sheep, cow, elephant, bear, zebra, giraffe

**Table 2:** Errors in the accuracy experiments.

	Time	Tokens	Tokens per Second	Tokens with Speed/Direction/Acceleration	Tokens with Calculation Error	Error Percentage
	32.19	100	3.107	94	6	6.000
	46.37	247	5.327	235	12	4.858
	38.74	128	3.304	122	6	4.688
	69.58	227	3.262	215	12	5.286
	35.18	111	3.155	110	1	0.901
	58.44	185	3.166	180	5	2.703
	51.98	233	4.482	221	12	5.150
	76.31	355	4.652	311	44	12.394
	40.39	130	3.219	126	4	3.077
	47.96	152	3.169	147	5	3.289
**Average**	**49.71**	**186.8**	**3.684**	**186.8**	**10.7**	**4.835**
